# Biosafety Data as Confidential Business Information

**DOI:** 10.1371/journal.pbio.1001499

**Published:** 2013-03-05

**Authors:** Kaare M. Nielsen

**Affiliations:** 1 Department of Pharmacy, Faculty of Health Sciences, University of Tromsø, Tromsø, Norway; 2 Genøk-Center for Biosafety, Science Park, Tromsø, Norway; King's College London, United Kingdom

## Abstract

Removal of confidentiality claims on biosafety data is necessary to adhere to standard scientific procedures of quality assurance, to increase transparency, to minimize impacts of conflicts of interests, and ultimately to improve public confidence in GMOs.

Confidential business information (CBI) is a necessary tool to protect commercial interests in the rapidly developing field of gene technology. CBI is also often claimed for documentation and materials supporting the biosafety assessments of genetically modified organisms (GMOs) intended for environmental release, food, and feed use. However, such claims oftentimes marginally serve their legitimate purpose to protect commercial interests and unnecessarily limit transparency and public peer review of data submitted to regulatory authorities. CBI and proprietary claims also restrict access to transgene sequence data, transgenic seeds, and other GMO materials, which precludes the development of independent research and monitoring strategies. In the long run, such claims are counterproductive to the safe and responsible commercial development of GM technology as they hinder the accumulation of biosafety data in the open, peer-reviewed literature, which is needed for both public and scientific consensus-building on safety issues and for improvements to the risk-assessment procedure itself. The increasing recognition of conflicts of interest as an invariable part of market-oriented safety-data production, interpretation, and risk communication also calls for transparency and open access to safety-related data and assessments.

## Biosafety Assessments

Biosafety assessments of GMOs, such as for crop plants intended for food and feed, are mandatory in most countries and required in international treaties such as the Cartagena Protocol on Biosafety. Most assessments and biosafety data are produced by the applicant and submitted directly to the regulatory authority together with the technical details of the product as well as responses to other requirements, often with portions labelled as CBI. Confidentiality claims are routinely made by companies in different fields to protect information that represents a harm or commercial disadvantage if known by business competitors. However, the CBI concept lacks a clear definition, and its use is based on the degree of claims made by the applicant. The extent CBI claims are used to prevent public disclosure of data needed to establish the safety of products entering the environment and the food chain therefore varies greatly between regulatory systems.

Biosafety data is produced by the applicant on a case-by-case basis and presented to regulatory authorities to support the commercial introduction of a specific genetic trait (event). In this way, most company-generated biosafety data is product specific and commercially relevant only to traits and cultivar combinations that the company owns and protects through patents and plant breeders' rights. Arguably, as patents provide exclusive rights for commercial use, most trait- and event-specific data on composition, environmental interactions, allergenicity, toxicity, and other safety aspects are of limited commercial utility to nonpatent holders. Moreover, such biosafety information cannot be used meaningfully for illegal product copying. Importantly, data protection can be offered independently of confidentiality. In the European Union (EU), regulation specifies that the information provided in a dossier cannot be used to the benefit of another applicant for a period of 10 years [1].

Unfortunately, lack of stringent standards, international harmonization, and transparency, as well as remaining claims of confidentiality on biosafety-relevant data generate consumer distrust, hinder public peer review, obscure handling of conflicts of interests, and place additional burdens on regulatory agencies as they must serve as the sole peer reviewers of extensive applications/dossiers. Moreover, CBI combined with intellectual property claims also control which actors can access GM crop material and thereby prevent the development of producer-independent biosafety research and monitoring strategies [2]–[5].

Here, I examine the justification of CBI claims on the data used to establish the safety of GMOs intended for commercialization and recommend major changes for the benefit of consumers, citizens, and GM developers.

## Lack of Standards for CBI Claims

There are no international guidelines, standards, or formal criteria related to what constitutes legitimate CBI claims in GM product applications [6]. Current acceptance of claims is based on past experience and established practice negotiated among risk managers and lawyers [7]. The basis and negotiations of confidentiality claims may be found in regulatory documents and case law. However, its historic and continual justification needs to be accessible also to the wider public. For the same GM product (event), the amount of an application claimed to be confidential varies between countries, and companies differ over the overall amount of information they consider confidential. This company practice seems at odds with the requirements for a legitimate purpose of CBI. For instance, the broad public disclosure of biosafety information present in GMO applications in New Zealand or Australia contrasts sharply with that available to the public in South Africa [8].

EU regulations provide for general public access to documentation and proscribe claiming some types of information as confidential, including the effects of the GMO food or feed on human and animal health and the methods for their detection [1],[9]. Although legal limitations to CBI claims are in place in some countries, access to data is not immediate due to delays, bureaucracy, and proprietary issues [10].

The lack of internationally accepted standards for defining legitimate CBI claims, and the resulting divergence in levels of disclosure between countries and companies, calls into question their commercial necessity and invalidates many confidentiality claims. Better alignment and improved communication among the staff of GM developers, including their innovation and safety researchers, regulatory assistants, lawyers, and communicators, may be needed to ensure that public interest and product recognition is placed ahead of CBI claims of limited commercial value. Several recommendations to improve CBI practices are outlined in Table 1.

**Table 1:** Some recommendations to improve the use of CBI claims on biosafety data.

1. National institutions/competent authorities can:	a) Develop stringent, uniform, and unambiguous policies on the types of information that can be labelled CBI.b) Ensure that all data (including raw data) related to the health and environmental impact of GMOs and products are exempted from CBI claims, and that legitimate CBI claims are strictly time-limited.c) Revisit institutional procedures currently in place to establish the validity of CBI claims and call for deliberations over what constitutes a commercial disadvantage, including a broad set of stakeholders.d) Initiate processes at the intergovernmental level (e.g., within the framework of the Cartagena Protocol on Biosafety) to ensure harmonization of CBI policies and practices.e) Require by law that the contiguous sequences of recombinant DNA in GM products approved for commercial release are made accessible to monitoring efforts by submission to open databases.
2. GM product developers can:	a) Develop company policies specifying that any part of data relevant to ensuring the health and environmental safety of a product is exempt from CBI claims. Such internal CBI policies should be harmonized across countries (and between companies).b) Avoid CBI claims on documentation or information available in patent descriptions.c) Improve access to commercially approved GM material for independent safety research so that material can be obtained freely, timely, and without legal conditionality. (It is noted that transboundary distribution of biological material may pose a challenge given asynchronous permits, legal responsibilities in case of spills, etc.).d) Aim to publish all biosafety data (in-house, outsourced, or from academic collaborations [53],[54]) used to support the safety of their marketed products within a reasonable time after commercialization, adhering to scientific standards, promoting transparency and avoiding unwarranted guest authorship and ghost-writing practices [55].e) Utilize the significant resource investment made in the preparation of data presentations for application dossiers, so as to limit additional workloads for publication.f) Contribute to international standardization of data presentation formats that can allow comparisons between studies. Standardized presentation allows raw data to be meaningfully published as supplementary material.g) Take initiative to publish meta-analyses of accumulated data in the peer-reviewed literature.
3. Research institutions can:	a) Benefit from the information made accessible for independent research. Identify areas where established knowledge possibly warrants less data collecting efforts in the future and areas with remaining uncertainty that warrant increased efforts. Such prioritization of research needs should involve relevant stakeholders to limit the potential for conflicts of interests to frame decisions on future research needs.b) Interact with GM developers to develop standardized data formats to enable robust meta-analysis and systematic reviews. Identify and develop systems for accessible, long-term data storage.

## CBI-Protected Biosafety Data Lacks Public Peer Review

**Figure 1:**
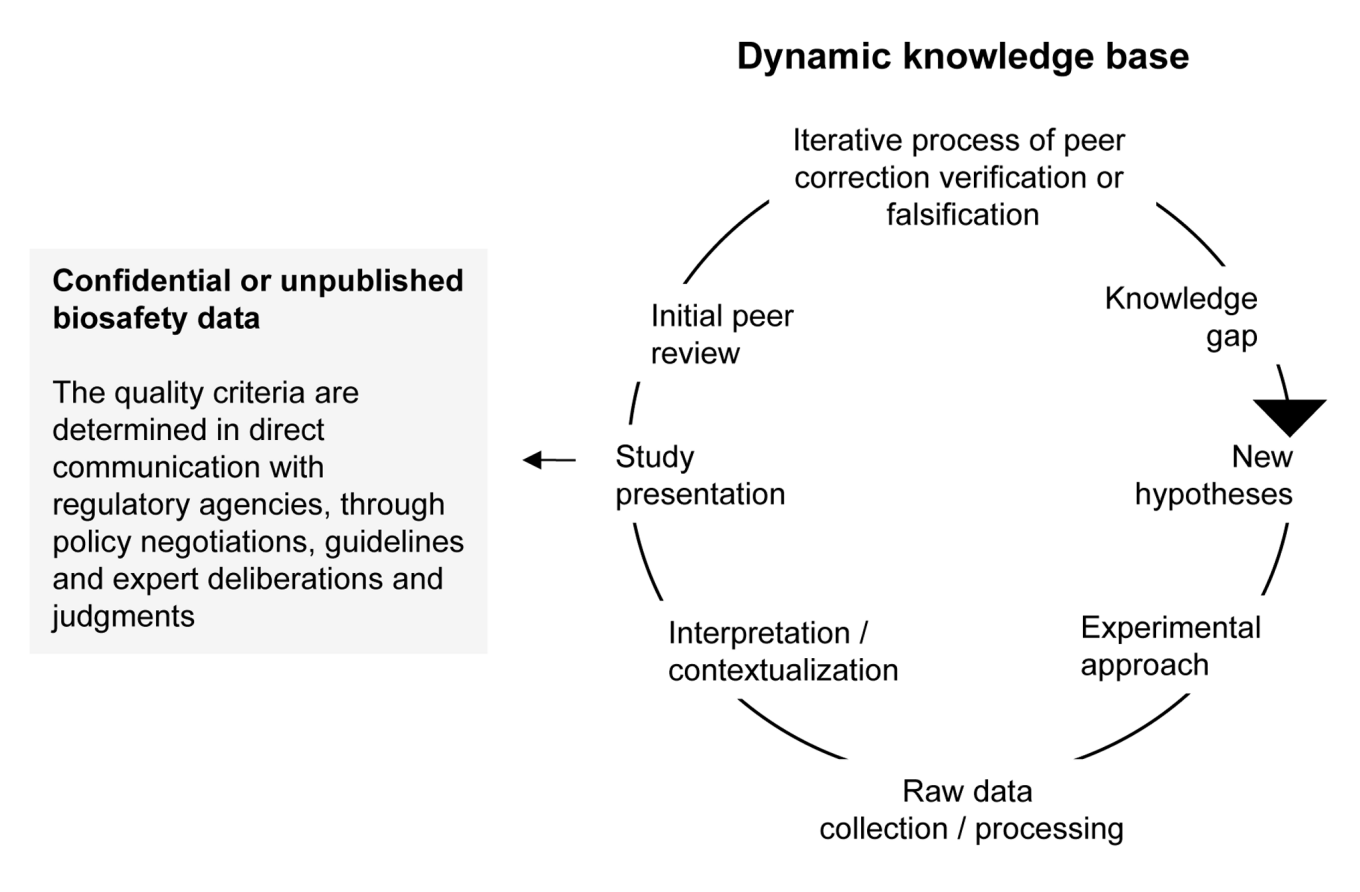
CBI-protected and other unpublished biosafety studies fail to adhere to the iterative process of knowledge production as they are not available for verification (resist falsification, reproducibility) and reevaluation by the open scientific community in light of new knowledge. Moreover, the knowledge produced by such studies does not accumulate in the publicly available scientific literature.

CBI claims prevent important biosafety data from entering the public domain of peer-reviewed science and accumulating in a dynamic knowledge base (Figure 1). Although the design of biosafety studies will be triggered by regulatory requirements rather than free inquiry, the generation of biosafety data does not differ from the general process of producing new scientific knowledge. Yet data necessary to assure the safe use of GMOs can be withheld from public peer review. On the positive side, commercial developers have now removed most CBI claims in regulatory documentation submitted to the EU. Moreover, the scientific review process of biosafety data offered by regulatory expert panels may substantially exceed that of some scientific journals. Nevertheless, regulatory expert panels cannot fully substitute for the quality assurance offered by broad collegial peer criticism of published studies, including the study design, the methods, the results attained, and their interpretation. By definition, sound scientific findings need to be subjected to and stand up to falsification, both before and after publication [11],[12]. Open peer access in product-oriented studies would allow conclusions to be reproduced, reevaluated, refined, and improved in light of continual improvements in methodology and biological insight [13],[14]. It is possibly a procedural risk factor in itself that reproducibility, transparency, and broad peer views are absent from the process in which the bulk of the “safety knowledge” is being produced and stored [15]–[17].

Science depends on peer review taking place both prior to and after publication. A timely example is the public and scientific debate following the publication of controversial biosafety studies [18]. Importantly, as illustrated in Figure 1, biosafety studies provided by the applicant may be exempt from similar public scrutiny due to confidentiality claims and or a general lack of efforts or incentives to make them available to open peer review. CBI-protected studies therefore do not contribute to the iterative scientific process and the knowledge obtained from them does not accumulate in the scientific literature. In general terms, CBI-protected studies fail to meet most established principles of knowledge production as articulated by Robert Merton's norms, including organized scepticism (e.g., open peer review), disinterestedness in study outcomes (e.g., absence of motivational bias), communism (e.g., open access to the scientific process), and universalism (e.g., standards) [19].

Data presented in application dossiers are already analyzed and presented in a form that is amenable to submission to regulatory agencies, decision-making, and communication (also in the presence of CBI claims). It is therefore possible to draw effectively on both the extensive efforts made by product developers and by experts from regulatory bodies for timely and resource-efficient online publication. For example, the written assessment made by the company, including raw data, methods descriptions, and data analyses, could be combined with the revisions called for by regulatory agencies and published in a dedicated online journal accessible to other scientists. Importantly, a well-defined electronic publication format will provide opportunities for standardized and contextualized datasets for meta-analysis [20]. Copyright issues on documentation assembled by the applicant will also need to be solved.

Although biosafety studies claiming “no observable adverse effects” may have previously been challenging to publish in peer-reviewed journals given editorial and peer-reviewer bias towards novel observations, this is arguably no longer the case if dedicated journals can be developed or expanded. Further developments in journal scopes could be envisioned with increasing demands for new standardized publication formats for biosafety studies. Moreover, the cost of printing is reduced by electronic formats and there are online storage opportunities for raw data as supplementary material.

## Conflicts of Interests in Biosafety Studies

Companies have market-oriented goals but are also required to produce the data, some under CBI protection, that support the safety of their products. This classic dilemma of perceived conflicts of interest arises at the intersections between environmental, health, and economic interests. The complexity of the GM events introduced, the biological variables investigated, regional environmental and socioeconomic differences, and the limited availability of uniform standards in study design and identification of relevant environmental variables make the experimental design and data-generating process vulnerable to conflicts of interests and bias [21]–[23]. For instance, lack of transparency may increase the potential for introducing bias in problem framing, methodological design, and statistical analysis of experiments, as well as for subjective data interpretation, contextualization, and selective data reporting [24],[25].

Outsourcing of biosafety studies to other commercial actors also generates possible sources of bias (e.g., funding source). Other researchers outside the commercial arena are also affected by the sources of motivation and bias described above [26]. Both the concepts of “independent” and “sound” science require further analysis for the degree of disinterestedness in study outcomes.

The close link between company-employed, outsourced, or collaborating researchers and the commercialization objectives of the product developer is a recurrent issue with no clear solution, except to adhere to scientific principles, recognized test standards, and integrity that can be maintained only through full transparency. Transparency in the choices and assumptions inherent in the design and interpretation of biosafety research is therefore a requirement for ensuring the quality of science used for policy and decision-making, especially because the criteria for knowledge production and selection in regulatory decision-making are unequal among stakeholders [27]–[29]. Further progress in international harmonization of minimal standards, methodological approaches, and publication formats may also be of immediate value for quality and robustness of studies [30]. Nevertheless, limitations in multilateral processes suggest most methodological guidelines will remain as voluntary provisions.

## Publication Bias in Biosafety Data

The availability of biosafety data in the scholarly literature reflects the motivation and resources invested in its production, release, and communication, rather than a neutral accumulation of objective, experimental observations made by disinterested scientists. It is counterproductive for a company to spend limited financial resources for academic analyses of products that would not end up in the marketplace. Arguably, the current industry practice of selective publication of safety data therefore contributes to a directional imbalance in the scientific production of new biosafety knowledge [31] because it encourages detailed dissection of published biosafety research, whereas unpublished or CBI-protected studies remain inaccessible for similar open scientific scrutiny [32],[33].

Financial and motivational constraints can therefore bias the flow of biosafety-relevant information to the scientific literature that shapes safety paradigms. The effects of such practices generate publication bias in product-oriented studies [34], and the bias seems further amplified by the lack of free access to GM seeds and other company-controlled research material for disinterested researchers/institutions.

## CBI Challenges the Capacity of Regulators/Regulatory Bodies

In most countries, governmental employees are responsible for the performance of regulatory risk assessment of GMOs in accordance with international and national laws. In the absence of mechanisms facilitating transparency and public peer review, regulators (or appointed part-time expert panels) must also, within strict deadlines, act as peer reviewers of the GMO producer's biosafety data and risk assessment (sometimes with external scientific input). Regulators/experts routinely review applications of up to several thousand pages that may contain substantial amounts of raw data and are required to issue decisions within a few months. Confidentiality in handling, storage, and processing of such applications represents a considerable resource investment that could partly be alleviated in the absence of CBI claims. CBI claims also place unnecessary limitations on openness in the risk-assessment process itself and can reduce public trust in regulatory bodies [35]. Moreover, the capacity to assess the necessity of CBI claims and perform adequate assessment on confidential biosafety data may be of particular concern in politically unstable and resource-limited countries [36],[37].

## CBI Claims with Conflicts of Interests Is a Generic Issue

Limited public access to safety data is not unique to GMO products but is embedded in all commercially driven research with proprietary goals. For instance, similar impacts of conflicts of interests, positive publication bias, limited transparency in decision-making, and restricted public access to clinical trial results are also recognized challenges in the field of pharmaceutical product development [38]–[42]. In this field, the potential for researchers to unwittingly influence study outcomes has been recognized and reduced by improvements in experimental design, e.g., double-blinded, placebo-controlled, randomized design trials and statistical power analysis, as well as other measures such as independent postmarket oversight and systematic reviews, upfront registration of randomized clinical trials, explicit data sharing, and full disclosure of interests. Despite the numerous actions taken, public overview, access to data, and transparency in the pharmaceutical field are far from achieved [43]–[49], though there is progress to increase transparency, openness, and reduce various types of bias [50]–[51].

I am not aware of similar initiatives to acknowledge sources of bias in market-oriented GM biosafety data production.

## CBI Claims Prevent Independent Research and Monitoring

The specific nucleotide-sequence data of the recombinant DNA constructs inserted into GMOs are generally not readily available to the scientific community due to confidentiality claims. Such claims lack functional ground since single sequences are available in the patent literature and can also be deduced from the larger construct presentations. In effect, only resource-limited, academic researchers ignorant of the patent literature and databases are hindered from easily accessing and deducing the exact DNA inserts. The limited electronic/database access to such technical GMO data remains troublesome for several reasons. First, it effectively limits detection of recombinant DNA to the company's specifications of primer binding sites, with embedded sensitivity and specificity, thereby preventing complementary monitoring protocols from being developed [52]. Second, the current regulatory requirements for detection methods may not facilitate the detection of mutated or fragmented recombinant DNA insertions because the detection methods rely on specific PCR primer sites provided by the GMO developer. Third, when relevant, it does not allow DNA sequences collected from environmental samples to be compared to commercialized recombinant DNA constructs (e.g., junction borders) through database searches.

DNA sequence comparisons could become an important tool to monitor unintended spread of novel recombinant gene constructs. A searchable DNA sequence database of all commercially released recombinant DNA constructs and insertion borders would significantly improve complementary monitoring approaches. A recombinant construct database would be of particular value for GM plants encoding non-food and feed traits, e.g., for the production of pharmaceuticals. Relevant authorities should review the limited access to insert sequence data and consider adopting regulatory statutes that require submission of contiguous recombinant DNA constructs to open-access databases prior to final GMO approval (Table 1).

## Summary and Recommendations

CBI is a necessary tool to protect commercial interests in the rapidly developing field of genetic engineering. I do not claim that company-sponsored and -conducted research is generally flawed or inferior to other sources of scientific knowledge, but rather challenge current practices and indiscriminate uses of CBI claims. Legitimate CBI claims should be reduced to those with significant commercial justification (Box 1). Moreover, although some regulatory systems explicitly prohibit CBI claims on biosafety studies, company practices may still make such studies unavailable to the wider public.

**Box 1. Warranted CBI claims, author's considerations:**

- Specific and substantiated CBI claims may be warranted on limited parts of the information present in GM applications when proof is provided that a significant financial investment has been made by the company, and the information is not generally known. A causal relationship between disclosure and harm should be provided. For example, time-limited confidentiality claims may be warranted for the details of the experimental protocols used for the DNA insertion/modification since such knowledge represents significant developmental costs and a clear competitive advantage.
- For information to be confidential, it must be known only to the claimant. Confidentiality is therefore not warranted on information present in patent documents or for information not considered to be or not under confidentiality agreements in other companies/locations/countries.
- Confidentiality is not warranted for data and studies collected through standard practices to establish product safety (e.g., following OECD and FAO/WHO guidelines). In practice, no experimental protocols or data from studies conducted with the purpose of demonstrating health and environmental safety should be claimed confidential. Study methods, techniques for biosafety data assembly, analysis, and interpretation should therefore be without CBI claims. Publicly available documents (e.g., use of published scientific literature) and assembled safety-assessment documents should not be considered confidential, although their initial collection and writing represents a cost. The public right of access to information on product safety outweighs such confidentiality claims.
- This contribution only discusses confidentiality claims made on data supporting the safety of commercialized/approved products. The disclosure of biosafety data in connection with experimental field introductions and exploratory trials are not discussed here and may require a quite separate approach to protect the intellectual, legal, and commercial interests of GMO developers in their early phases of product development.

Data production for regulatory purposes remains stuck in a closed frame, against the trend of openness in data access and sharing emerging in other scientific fields. Change is needed from a culture of secrecy, caused by CBI claims and limited disclosure of proprietary data, to transparency, openness, and adherence to standard principles of knowledge production. Consumer distrust is a natural outcome when independent analyses are absent from the process in which knowledge is produced and verified. Moreover, making biological material largely inaccessible for independent research is counterproductive both to science and to building public trust as well as further GM market developments. Availability of peer-reviewed studies and standardized publication formats could also nourish a more constructive and scholarly discussion of the risk relevance of the extensive sets of data routinely collected.

## Abbreviations

CBI: confidential business information
EU: European Union
GMO: genetically modified organism
